# Brief Report: Quantifying Speech Production Coordination from Non- and Minimally-Speaking Individuals

**DOI:** 10.1007/s10803-023-06206-0

**Published:** 2024-04-13

**Authors:** Tanya Talkar, Kristina T. Johnson, Jaya Narain, Pattie Maes, Rosalind Picard, Thomas F. Quatieri

**Affiliations:** 1 Human Health and Performance Systems, MIT Lincoln Laboratory, Lexington, MA USA; 2 Speech and Hearing Bioscience and Technology, Harvard Medical School, Boston, MA USA; 3 MIT Media Lab, Massachusetts Institute of Technology, Cambridge, MA USA; 4 Department of Electrical and Computer Engineering, Northeastern University, Boston, USA; 5 Department of Communication Sciences and Disorders, Northeastern University, Boston, USA

**Keywords:** Minimally verbal, Affective state, Emotions, Speech production, Autism spectrum disorder

## Abstract

**Purpose:**

Non-verbal utterances are an important tool of communication for individuals who are non- or minimally-speaking. While these utterances are typically understood by caregivers, they can be challenging to interpret by their larger community. To date, there has been little work done to detect and characterize the vocalizations produced by non- or minimally-speaking individuals. This paper aims to characterize five categories of utterances across a set of 7 non- or minimally-speaking individuals.

**Methods:**

The characterization is accomplished using a correlation structure methodology, acting as a proxy measurement for motor coordination, to localize similarities and differences to specific speech production systems.

**Results:**

We specifically find that frustrated and dysregulated utterances show similar correlation structure outputs, especially when compared to self-talk, request, and delighted utterances. We additionally witness higher complexity of coordination between articulatory and respiratory subsystems and lower complexity of coordination between laryngeal and respiratory subsystems in frustration and dysregulation as compared to self-talk, request, and delight. Finally, we observe lower complexity of coordination across all three speech subsystems in the request utterances as compared to self-talk and delight.

**Conclusion:**

The insights from this work aid in understanding of the modifications made by non- or minimally-speaking individuals to accomplish specific goals in non-verbal communication.

**Supplementary Information:**

The online version contains supplementary material available at 10.1007/s10803-023-06206-0.

## Introduction

Non-verbal vocalizations, such as grunts, yells, squeals, moans, and babbles, are typically used in social contexts to provide additional information in communication. These vocalizations help indicate emotional state or functional information independent of words with linguistic meaning (Hall et al., 2019). Individuals who are non- or minimally speaking, however, may rely heavily on non-verbal vocalizations to communicate intent or emotion, supplementing a repertoire of zero to a handful of words or word-like approximations (Koegel et al., 2020). While these non-verbal communications may be easily understood by caregivers or those close to the individual, unfamiliar individuals may find it difficult to comprehend the subtleties present in the vocalizations. There has been little research into characterizing and understanding the non-verbal vocalizations of the non- or minimally-speaking population, who have been referred to as non- or minimally-verbal individuals in the past. This population includes approximately 30% of individuals with autism spectrum disorder (ASD) as well as persons with certain genetic disorders or neurological differences (Tager‐Flusberg & Kasari, 2013; Vrečar et al., 2017). A better characterization and understanding of non-verbal vocalizations across multiple communication intents may help caregivers and strangers to interpret vocalizations from non- or minimally-speaking individuals.

Some prior research on non-verbal vocalizations has focused on identifying acoustic properties and emotions conveyed in communications produced by neurotypical individuals. For example, one study determined that emotions conveyed in non-verbal vocalizations, such as ‘anger’ and ‘disgust’, could be interpreted by individuals from multiple cultural backgrounds (Sauter et al., 2010). Non-verbal vocalizations have additionally been utilized to help predict emotions portrayed in speech by developing machine learning models trained on the vocalizations. One such model led to an accuracy of 0.61 (Hsu et al., 2021) and another a correlation of 0.73 between actual and predicted emotions (Xin et al., 2022). Both of these studies predicted emotion categories such as ‘Anger’, ‘Sadness’, ‘Happiness’, ‘Fear’, and ‘Excitement’ using sounds produced by neurotypical adults across multiple languages. Another study developed an automated processing tool for non-verbal vocalizations in babies or toddlers which aided in understanding and predicting language delays (Oller et al., 2010). Additional research has focused on pre-verbal vocalizations in infants. One such study identified specific acoustic changes that occurred in vocalizations as infants aged (Scheiner et al., 2002). Another study showed that non-caregiver listeners were able to almost perfectly identify positive or negative emotion conveyed in vocalizations from pre-verbal infants, though they may have not been able to detect subtleties that existed within positive or negative categories, such as discriminating between ‘Pain’ and ‘Demand for Food’ (Lindová et al., 2015). While the studies highlighted above show the utility of non-verbal vocalizations in emotion recognition, the datasets in these studies have limited or no labeling of communicative functions of the non-verbal vocalizations, such as ‘request’. Additionally, the labels cannot be easily translated over to the vocalizations of non- or minimally-speaking individuals as there needs to be a wider range of labels to cover the communicative intent of these individuals. This motivates the need for studies and datasets that are focused specifically on non- or minimally-speaking individuals. This pilot study analysis is part of a larger study which aims to classify and characterize the speech of non- or minimally-speaking individuals, focusing on categories of vocalizations (e.g., frustrated or delighted) that have been labeled by caregivers (Johnson et al., 2023; Narain et al., 2020).

**Fig. 1:**
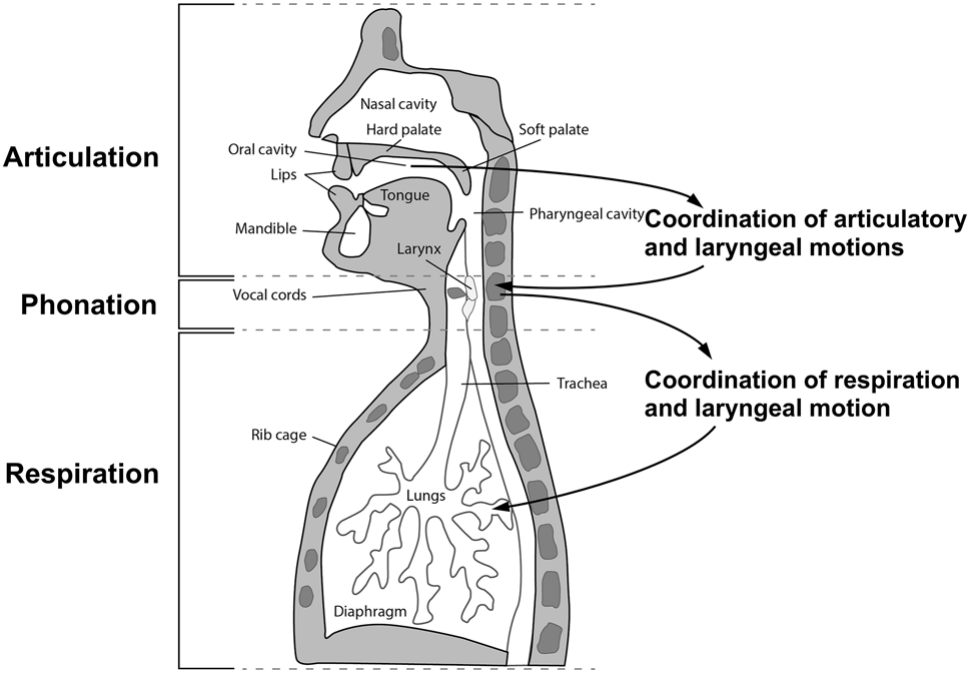
Depiction of the three main speech production subsystems and their interactions. Coordination of movements happen both within and across speech production subsystems

In this work, we utilize a novel correlation-based methodology that has been developed and used to predict the presence of and characterize speech production motor challenges in individuals with ASD (Talkar et al., 2020a, 2020b), Major Depressive Disorder (Williamson et al., 2019), Traumatic Brain Injury (Talkar et al., 2021), and Parkinson’s Disease (Smith et al., 2018). The features describe the relative coupling or independence of movements within and across three speech production subsystems—articulatory, laryngeal, and respiratory. The physiology of and the interactions between these speech production subsystems are depicted in Fig. 1. The articulatory subsystem is comprised of the vocal tract and articulators within it, such as the tongue, jaw, and lips. The laryngeal system contains the larynx, which house the vocal folds and surrounding musculature. The respiratory system involves the lungs and breathing mechanisms that allow for airflow through the larynx and subglottal pressure. Speech production, both verbal and non-verbal, requires coordination of the timing of movements within and across all three subsystems, which can be measured, by proxy, by correlating acoustic time series representative of each subsystem. For example, in the articulatory subsystem, the jaw moving forward and the lips opening for the sound ‘ee’ need to occur at a certain relative timing to generate the right sound. We can measure deviations from or adherence to this relative timing utilizing the correlation-based methodology.

Characterizing the differences in subsystem coordination between non-verbal vocalization categories may aid in understanding how vocalization classes, such as ‘request’ or ‘delight’, are modulated in speech production subsystems to communicate different emotions or to serve different functions across non- or minimally-speaking individuals. This can, in turn, be used to create machine learning models to automatically interpret vocalizations from non- or minimally-speaking individuals for people who do not know them or are getting to know the individual. Clinicians could additionally utilize the speech motor coordination characterizations to understand how the vocalizations of non- or minimally-speaking individuals are changing over time, or to help determine the need for speech therapeutic trajectories that could improve the interpretability of non-verbal vocalizations in these individuals.

## Methods

### Participants

Participants were recruited for this initial pilot study through conversations with community members and word-of-mouth. Eight participants provided sufficient samples for further analysis with > 50 labeled vocalizations for at least 4 categories. All vocalizations from these participants are included in the online database, ReCANVo (Johnson et al., 2023). These participants ranged in age from 6 to 23 years and had one or more parent-reported diagnoses of ASD, cerebral palsy (CP), and genetic disorders. They all had fewer than 10 spoken words or word approximations, as reported by caregivers. No other exclusion criteria were enacted to be able to capture as many individuals as possible under this unique and understudied population. Details about the subjects and their demographics are provided in Table 1, and additional demographic details as well as information about specific alternative and augmentative communication (AAC) use are listed in Johnson (2021). A diverse set of diagnoses were utilized to ensure analysis of individuals across the non- or minimally-speaking spectrum, with the intent of studying commonalities and differences across the different causes with the potential of developing clusters or phenotypes of the non- or minimally-speaking condition in future work. The study was approved by the Massachusetts Institute of Technology Committee on the Use of Humans as Experimental Subjects Institutional Review Board. Informed consent was obtained for all participants in the study. (Table 1).

**Table 1:** Demographic details of the seven participants who were included in the analysis

Subject ID	Age	Gender	Diagnosis	Number of spoken words	AAC use?
P01	18	M	ASD, Down Syndrome (DS)	0	Yes
P02	19	M	ASD	4	No
P03	9	M	ASD, rare genetic disorder	0	Yes
P05	11	F	ASD	0	No
P06	12	M	CP	3	Yes
P08	6	F	ASD	0	Yes
P11	10	M	CP	1	No
P16	7	M	ASD	5–8	Yes

### Recording and Segmentation

Audio samples were collected using a wireless recorder (Sony IDC-TX800; 16-bit, 44.1 kHz stereo) attached to the participant’s clothing using strong magnets for P01, P02, P03, P06, P11, and P16, worn as a necklace for P08, or placed nearby for P05. This process took into account any tactile sensitivities the subject had. The caregiver, acting as the labeler, utilized a custom Android application to label the start and end of vocalizations the participant made in a natural environment (e.g., the participant’s home) in real time. Caregivers were asked to go about their day-to-day activities while recording to capture a variety of scenarios at their convenience and maximize the types of vocalizations provided by the participant. Though this practice resulted in variability in data collection and labeling practices by participants, it was critical in conducting this novel data collection process for vocalizations of non- or minimally-speaking individuals.

Six of the vocalization labels in the application were constant across all of the subjects—self-talk, delight, dysregulation, frustration, request, and social exchange. The labels were defined as follows:

- Self-talk: Vocalizations that are made to one’s self, without any other obvious/overt communicative meaning or intent. These are typically made without an obvious trigger, and could be associated with a generally regulated, relaxed, or content state.
- Delight: Vocalizations that appear to be associated with excitement, glee, and happiness.
- Dysregulation: Vocalizations that appear to be associated with being irritated, upset, agitated, bored, uncomfortable, or distressed. These vocalizations are often made without an apparent trigger or communicative intent to an outside observer. However, the dysregulated state may be due to overstimulation, an altered sensory or internal bodily state, or an event that an outside observer cannot immediately control or alter and present as a negative affect state.
- Frustration: Vocalizations that appear to be associated with being frustrated, angry, or protesting. These vocalizations are usually caused by a specific situation and carry communicative intent, distinct from the cause of a dysregulated state. Potential triggers include the inability to achieve an objective or complete a task.
- Request: Vocalizations that carry the communicative intent of making a specific request (e.g., for food or an item).
- Social Exchange: Vocalizations that appear to be social in nature (e.g., as part of a back-and-forth vocal exchange while playing a game).

Distinctions of these categories were discussed with the caregivers, and they agreed to the labeling schema and definition of the vocalization categories. Further details about the definition of the labels provided as guidance to caregivers is given in Narain et al., 2022 and Johnson et al., 2023. Labelers were walked through the smartphone application during an intake call to become familiar with the labeling, and were instructed to only label vocalizations that they felt confident they understood and had an associated label on the app. Labelers additionally were asked to utilize contextual clues to determine labels, such as environment, body movements, facial expressions, and the presence of other individuals, though these were not recorded. There was also an option to add in 4 custom labels from a list of 25 pre-selected affective or communicative functions. The participating families periodically uploaded the audio data to an IRB approved cloud-based service, while the labels from the app were automatically synced to a server.

The segmentation process first isolated periods of interest in the audio around the caregiver’s label, taking into account human delays in labeling. Then, each segment was verified manually to eliminate recordings with overlapping voices and trim extraneous noise and surrounding vocalizations. This led to over 7000 segments. See Johnson et al., 2023 for full details of this process. For the analysis in this paper, segments of length less than 2.5 s were additionally eliminated to allow for sufficient data within each file. Due to a lack of audio segments labeled as ‘dysregulated’ from one of the subjects (P02), that subject was not utilized in further analysis, and therefore the results include the data from 7 total subjects. Additionally, given the small number of samples in the social exchange category as well as the potential overlap between the social exchange category and the other classes, only samples from self-talk, delight, dysregulation, frustration, and request were used in analysis. A total of 5670 recordings were processed in this analysis. Table 2 depicts the subjects and the number of recordings analyzed in each category as well as the number of weeks over which data was collected.

**Table 2:** Number of vocalizations collected for each subject in each of the categories analyzed as well as number of weeks over which data was collected

Subject ID	Dysregulated	Frustrated	Self-talk	Request	Delighted	Timespan of data (weeks)
P01	212	150	564	130	357	64
P03	302	47	55	61	25	56
P05	116	283	286	6	235	11
P06	5	30	56	124	227	4
P08	13	781	503	44	39	20
P11	22	27	33	22	207	19
P16	34	162	354	19	139	10

### Low-Level Features

A set of 9 acoustic and articulatory features were extracted from each audio segment to represent three speech production subsystems—articulatory, laryngeal, and respiratory. These features are derived from the speech signal to represent basic temporal or spectral characteristics of the acoustic measurement. The articulatory subsystem was represented by formants (vocal tract resonances), mel-frequency cepstral coefficients (MFCCs), and articulatory tract variables (TVs). We extracted the first three formants (F1–F3) at a sampling rate of 100 Hz using the Kalman-based autoregressive moving average (KARMA) software (Mehta et al., 2012). The formants represent the positioning of articulators, such as the tongue and lips, in the vocal tract, which can be moved to specific configurations for production of consonant and vowel sounds. MFCCs were extracted at a sampling rate of 200 Hz using the Praat software (Boersma & Weenink, 2018). MFCCs also reflect articulator movement and positions within the vocal tract as measured through changes in the spectrum. We used an acoustic-to-articulatory inversion software to extract six TVs at a sampling rate of 100 Hz, representing positions of the articulators in the vocal tract (Sivaraman et al., 2019). TVs represent a more physiologically accurate representation of articulator positions and movements, such as where and how close the tongue body or tongue tip is to the top of the mouth.

The laryngeal and respiratory subsystems were represented by features capturing voice quality, prosody, and loudness. Fundamental frequency (F0) and harmonic-to-noise ratio (HNR) were extracted at sampling rates of 1000 and 100 Hz respectively using the Praat software (Boersma, 1993; Boersma & Weenink, 2018). F0 represents the frequency of vibration of the vocal folds within the larynx, which leads to higher or lower pitch of the vocalization. HNR represents the degree of turbulent air created through and along the edges of the vocal folds that is let through the opening between the vocal folds (referred to as the glottis). A lower HNR means that a higher volume of air is passing through, which can lead to the perception of a breathy voice. Cepstral peak prominence (CPP) and creak were extracted at a sampling rate of 100 Hz using custom MATLAB scripts (Fraile & Godino-Llorente, 2014; Heman-Ackah et al., 2002). CPP represents the ‘regularity’ of vocal fold vibrations, with a higher value indicating that the vocal folds are vibrating at a constant speed as opposed to a varying speed over time. Creak is a voice quality measure that represents how slack the vocal folds are during vibration. If the vocal folds are very slack, there is less airflow that passes through the glottis, and the resulting voice sounds ‘creaky’ or ‘scratchy’, which is typically seen in elder individuals. The speech envelope, an approximation of the respiratory subsystem, was extracted at a sampling rate of 100 Hz using a custom MATLAB script (Horwitz-Martin et al., 2016). Speech envelope represents loudness of an individual’s voice. All features and a summary of their physiological and perceptual interpretations are laid out in Table 3. All acoustic and articulatory features were extracted as time series, reflecting the trajectory of the features over the course of each audio recording.

**Table 3:** Low-level features extracted from acoustic signals and their physiological origin as well as their effect on speech perception

Feature	Physiology	Perception
Formants	Vocal tract articulator movements	Defines consonant and vowel perception
MFCCs	Vocal tract articulator movements	Derived from spectrum of speech
Fundamental frequency (F0)	Vocal fold vibrations	‘Pitch’ of speech
Harmonic-to-noise ratio (HNR)	Noise at the glottis	Hoarseness
Cepstral peak prominence (CPP)	Stability of vocal fold vibrations	Erratic pitch
Creak	Compression of vocal folds due to movement in the surrounding musculature which cause the vocal folds to become slack during vibration	Voice sounds like a creaky door, also referred to as vocal fry
Speech envelope	Contributions of the respiratory system and resonance-harmonics interaction to modulate amplitude of speech	Reflected in intensity or loudness of speech, as well as in prosodic features such as rhythm and stress patterns

These features were utilized to generate high-level features representing motor coordination

### High-Level Features

**Fig. 2:**
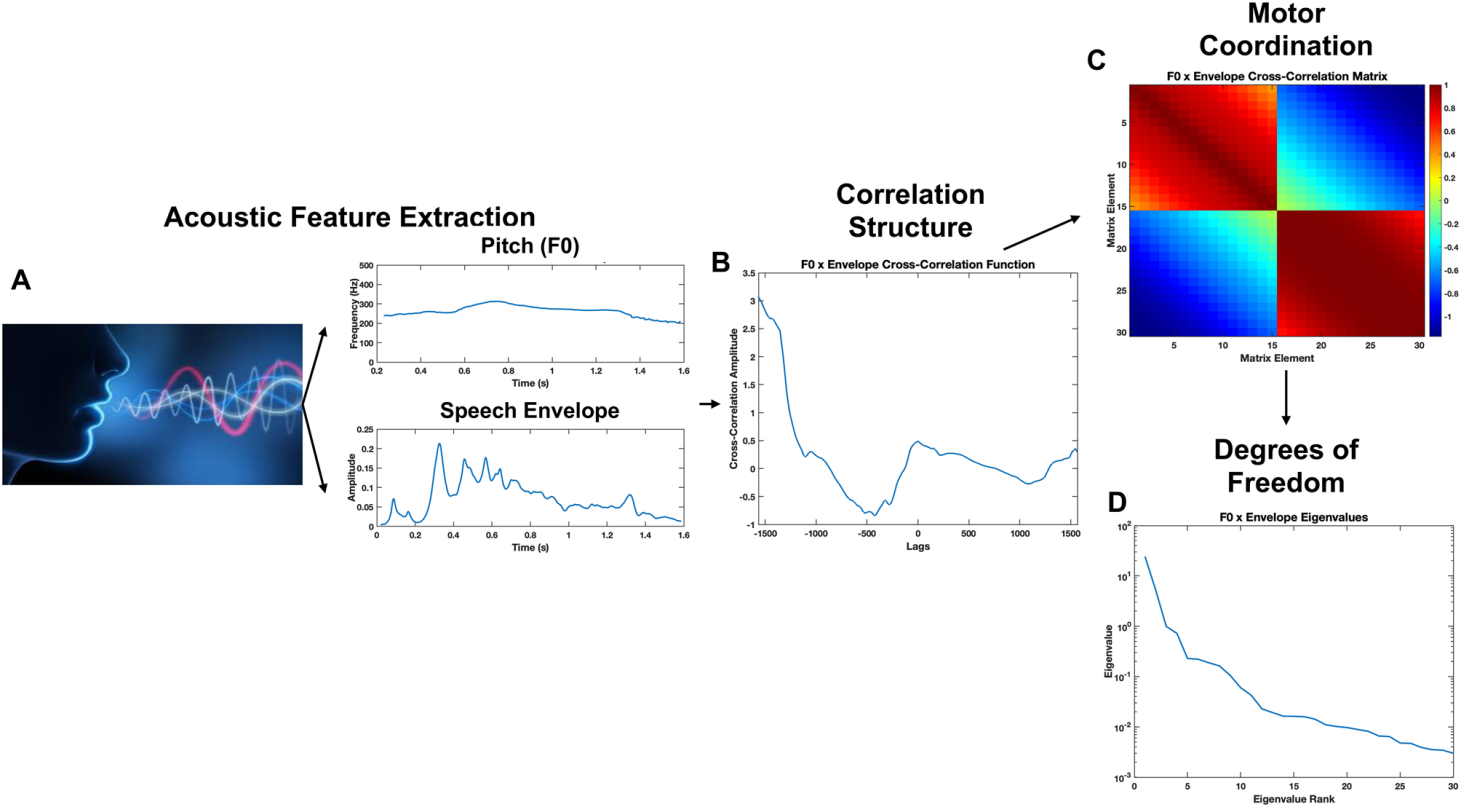
Example of the cross-correlation matrix methodology utilizing F0 and speech envelope. Because of how air flow and pressure from the lungs influences the vocal fold vibrations, F0 and speech envelope are known to change in a coordinated fashion in neurotypicals. This coordinated movement can be thought of as a dynamical system with some number of degrees of freedom. **A** To derive the number of degrees of freedom from a recorded vocalization, we first obtain the fundamental frequency, or pitch, (F0, top) and speech envelope (bottom) of each vocalization. An example extraction from a frustrated vocalization from P05 is shown here. **B** Then, we compute the cross-correlation function of F0 and the speech envelope, where the value at each lag represents the correlation of the signals moved relative to each other by one sample. **C** The correlation matrix consists of off-diagonal blocks that contain samples from the cross-correlation function of F0 and speech envelope. The diagonal blocks reflect samples of the auto-correlation function of F0 and speech envelope, each. **D** Finally, we compute the eigenvalues of the correlation matrix, which can then be compared to the eigenvalues from other vocalization labels to determine the relative coupling or independence of the F0 and speech envelope signals. The eigenvalues are ranked from greatest to smallest to compare across vocalization categories

**Fig. 3:**
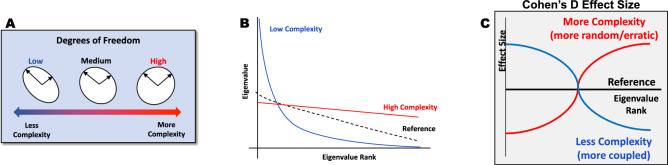
Eigenvalues extracted from correlation matrices can be compared by calculating a Cohen’s d effect size for each eigenvalue (ranked from greatest to smallest) across two vocalization categories. This trajectory of the effect sizes across the ranked eigenvalues determines relative complexity of the underlying signals based on the degrees of freedom needed to explain the majority of the variability. Low complexity, or higher coupling between the underlying signals, is reflected in a few larger eigenvalues of low rank and many smaller eigenvalues, which would be reflected in a positive to negative pattern in effect sizes across eigenvalue rank when compared to signals of higher complexity. On the other hand, high complexity, or more independence between the underlying signals, is reflected in eigenvalues that are of similar magnitude to each other, which would lead to a negative to positive pattern in effect size across eigenvalue rank when compared to signals of lower complexity

Our goal with high-level features is to give insight into the nature of the dynamical motor systems that underlie the movements within and across the three subsystems. We view these dynamical systems with respect to their *degrees of freedom*. For example, systems with highly coupled components, such as when the lips and jaw move together at similar amplitudes and timings, have few degrees of freedom, and we refer to these systems as having low complexity. On the other hand, systems with many independent moving components, such as the tongue moving independently from the lips and jaw to help quickly produce consonants, have many degrees of freedom, or high complexity. We can estimate these degrees of freedom by a method known as time-delay embedding, which entails the formation of correlation matrices. These matrices are filled with samples of auto- and cross-correlations of the low-level acoustic and articulatory time series. Auto-correlations refer to correlations computed between a time series and itself, while cross-correlations refer to correlations computed between one time series and another time series. This process is described mathematically in previous work on correlation structures (Talkar et al., 2020a, 2020b; Williamson et al., 2019) and is depicted in Fig. 2 with an example of the cross-correlation of F0 and speech envelope. We derive insights into the relative complexity of the underlying signals from the eigenvalues of the correlation matrices, as shown in Fig. 3. Eigenvalues represent the degrees of freedom required to describe the system represented by the signals that make up the correlation matrix. The sum of the magnitude of the eigenvalues is the same as the size of the correlation matrix (e.g., a correlation matrix of size 15 × 15 means that the sum of the eigenvalues must be 15). Therefore, the distribution of the magnitude of the eigenvalues can provide insight into the complexity of the underlying system (Hadd & Rodgers, 2020).

Higher complexity of a feature or across multiple features is represented in a more uniform distribution of eigenvalues. For example, a correlation matrix of size 15 × 15 could yield eigenvalues all with the value of 1, which represents high complexity. Lower complexity, however, is represented through a larger proportion of the overall signal variation being concentrated in a small number of eigenvalues, such as a correlation matrix of size 15 × 15 that has one eigenvalue with a magnitude of 8 while the others are of magnitude 0.5. Higher complexity in a set of signals manifests as a combination of higher frequency content, phase shifts between signals, or noise added to signals (Talkar, 2023). Additional details and examples of high complexity signals are provided in the Supplementary Materials. Higher complexity can result from signals representing movements that are more random or erratic relative to each other, such as the comparison of slower movements of the articulators in the vocal tract to the rapid movements of the vocal folds during speech. In other words, these movements are largely decoupled from each other, resulting in a more complex interaction. Lower complexity can result from signals representing movements that are tightly coupled across time, such as the lip and jaw moving together during speech production. We can therefore compare the relative complexity of individual and combinations of acoustic and articulatory features across multiple communication classes to understand how speech motor coordination differs between the classes.

Correlations across features were calculated within and across speech subsystems for a total of 18 feature combinations. When correlations of features related to the laryngeal subsystem were calculated, a masking technique was utilized to include only those speech regions when a non-zero pitch is detected by the Praat pitch estimator—i.e., only time periods when the vocal folds are vibrating. When calculating auto-and cross-correlations, we computed 15 different correlations, starting with the signals correlated with 0 ms time delay, then with 10 ms time delay, 20 ms time delay, and so on with 10 ms time delay steps until we reached 15 correlations. This allowed us to collect the samples to fill in the correlation matrix as shown in Fig. 2. Each feature combination resulted in 15 * *n* eigenvalues, where *n* is the number of signals input to form the correlation matrix.

After they were extracted from correlation matrices, eigenvalues were ranked from greatest to smallest. Cohen’s d effect sizes of the ranked eigenvalues were computed across different vocalization categories to capture the relative complexity of the feature in one vocalization category compared to another (Diener, 2010). To compute the group effect size patterns, we created correlation matrices for each recording belonging to a subject and extracted the eigenvalues from each correlation matrix. The resulting eigenvalues across all recordings within a particular class for each subject were averaged to prevent bias from subjects with more data. The averaged eigenvalues for all 7 subjects for one class were then compared to the averaged eigenvalues for all 7 subjects for a second class by computing the Cohen’s d effect size, subtracting Category 2 from Category 1. We refer to the shape that is formed by the Cohen’s d effect sizes when looking across the eigenvalues as the ‘effect size pattern’. The effect size patterns provided insights into relative complexity of a particular feature for one vocalization category as compared to another, as indicated in the left-most panel of Fig. 3.

Specifically, a pattern of positive-to-negative effect size (or high-to-low) indicates that the eigenvalues from Category 1 were more concentrated in a group of a low ranked eigenvalues as compared to those from Category 2, meaning that there is higher complexity in Category 1, or more degrees of freedom. Lower complexity in Category 1 manifests as a negative-to-positive effect size (or low-to-high). The effect size patterns of comparisons of categories within individuals were also calculated to see how the group level effect size pattern compared to those of the individual subjects. Patterns that showed a clear low or high complexity effect size pattern were selected for pattern analysis across the categories as these were eigenvalue effect sizes with concentrated spread and thus interpretable to determine lower or higher complexity between classes (Figs. 2 & 3).

## Results

In this section, we will compare various sets of non-verbal vocalization categories. First, we compare frustrated vocalizations to dysregulated vocalizations, as these categories portray a similar emotion, yet are caused by different triggers. The other categories, request, self-talk, and delight, are then compared to frustrated and dysregulated, as we are interested to see whether the similarities in the latter categories will manifest in comparisons to the first three categories, representing varied emotional and functional categories. Finally, we compare request, self-talk, and delight to assess what speech production subsystems contribute to differences in these categories.

### Frustrated vs. Dysregulated

**Fig. 4:**
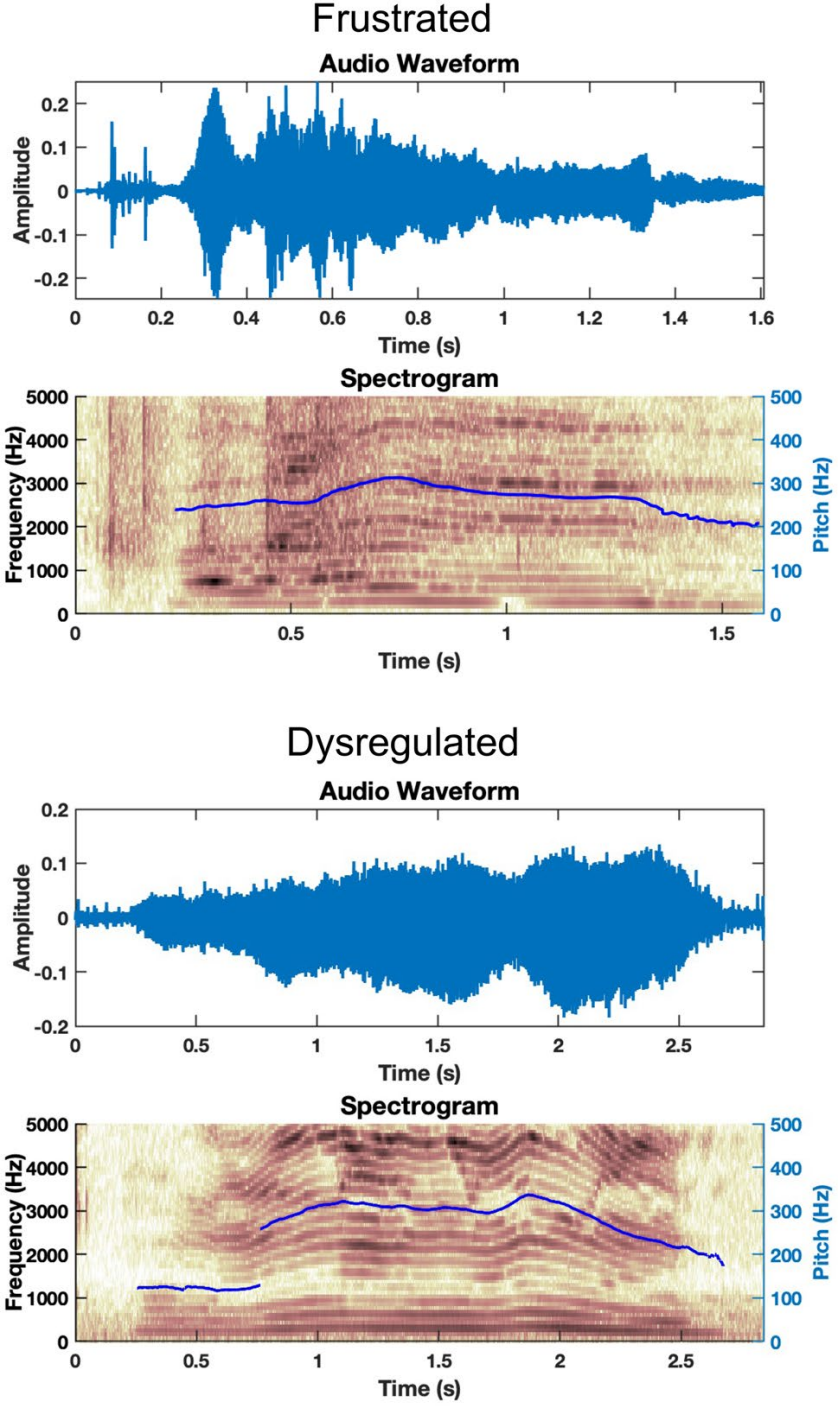
Spectrograms depicting frustrated and dysregulated vocalizations from a single subject (P05). The F0 trajectory (blue line) in the dysregulated vocalization has a higher complexity due to higher frequency content in the trajectory. More complex pitch in the dysregulated case is reflected in more variability not only in the pitch itself but also amplified variability in high harmonics

**Fig. 5:**
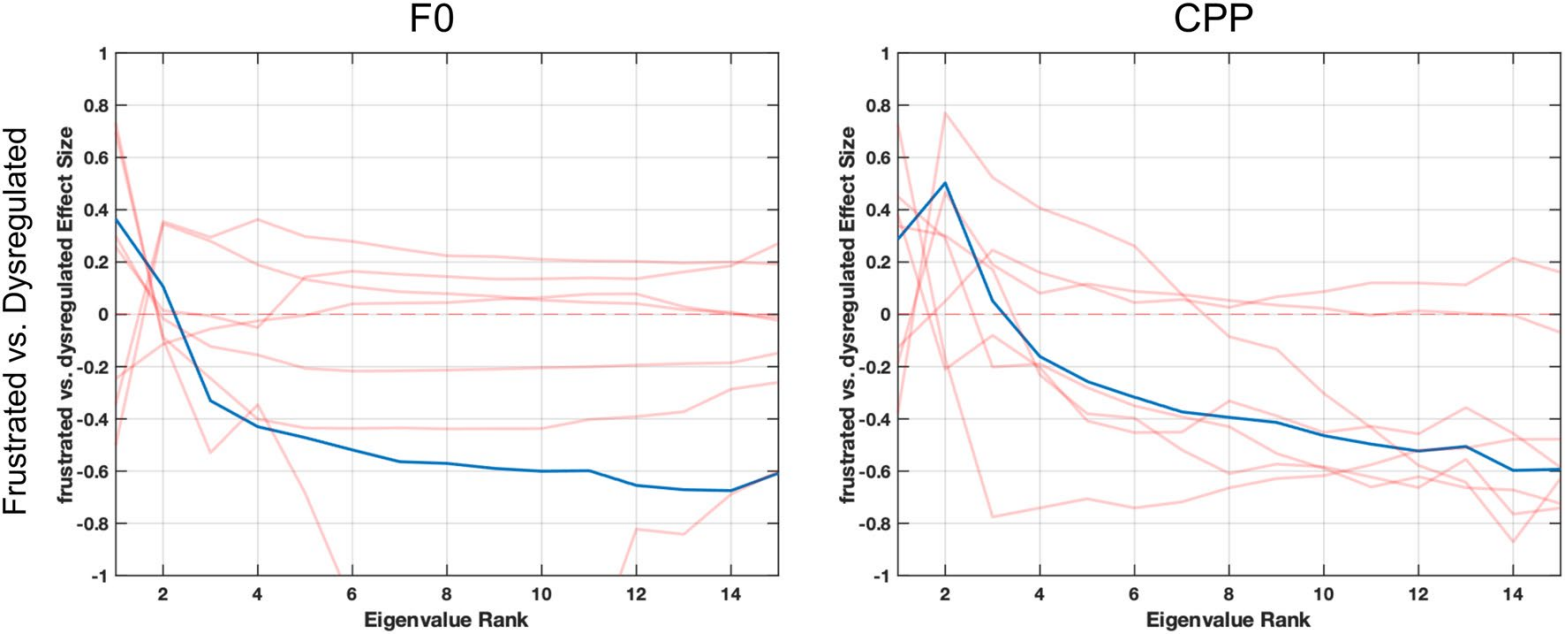
Cohen’s d effect size patterns for eigenvalues comparing frustrated and dysregulated vocalizations for F0 and CPP. Blue lines indicate group-level comparisons. Red lines indicate the effect size patterns of individual subjects. Plots indicate that frustrated vocalizations are often less complex than dysregulated vocalizations in the laryngeal subsystem as indicated by the positive to negative pattern seen in eigenvalues across the ranked eigenvalues

In differentiating between frustrated and dysregulated utterances, frustration is typically triggered by a specific event, such as not being able to go outside due to rain or the inability to fix an ACC device, versus dysregulation which might not be tied to a specific event that can be altered by an observer, but rather, as an example, might be due to not getting enough sleep, experiencing constipation, or having a headache. Figure 4 depicts spectrograms that highlight differences in frustration and dysregulation vocalizations for a single subject (P05), showcasing a higher degree of pitch modulation in the dysregulated vocalization (bottom) as compared to the frustrated vocalization (top). Any gaps in the pitch contours in the spectrogram are due to the software Praat being unable to detect vocal fold vibration in those frames, and thus those regions are not included in analysis. Figure 5 depicts the effect size patterns for features which showed clear pattern differences between the two categories. Differences in physiology appeared to be localized to the laryngeal subsystem, corresponding to strong effect size patterns for laryngeal-based acoustic features. Lower complexity of movement was seen in trajectories of F0 and CPP in frustrated vocalizations versus dysregulated vocalizations when compared at the group level, as portrayed by the positive-to-negative effect size patterns. No clear patterns of low or high complexity were seen in articulatory- or respiratory-based features at the group level, or in correlations across subsystems, with effect sizes being small in these comparisons (Figs. 4 & 5).

### Other Categories vs. Frustrated or Dysregulated

**Fig. 6:**
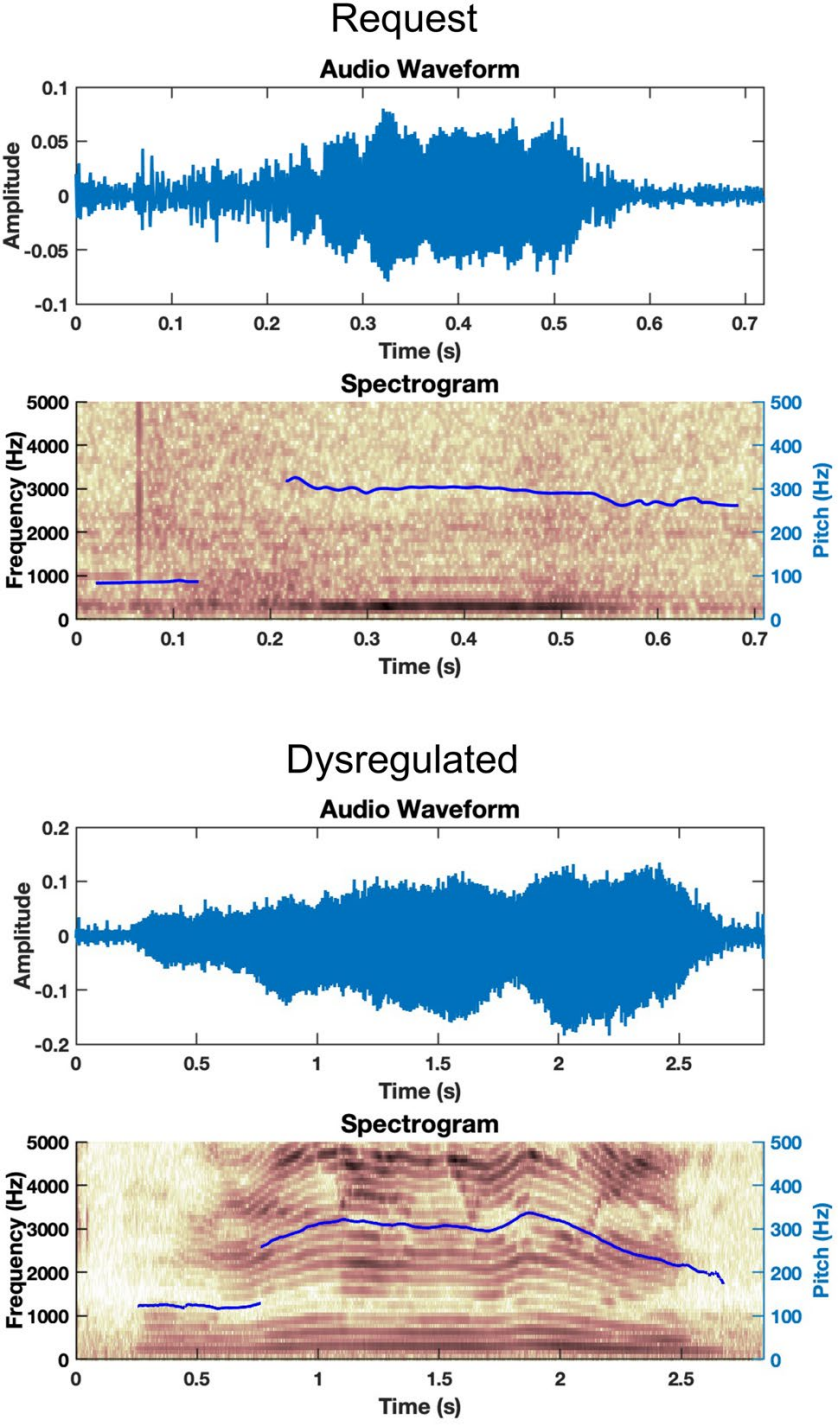
Spectrograms depicting request and dysregulated vocalizations from a single subject (P05). For the request utterance, we see a sustained pitch contour. However, there is modulation of F0 in the dysregulated case

**Fig. 7:**
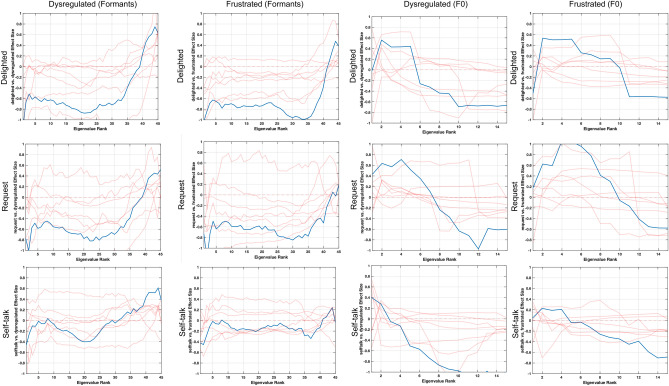
Cohen’s d effect sizes comparing eigenvalues from delight, request, and self-talk vocalizations to dysregulated and frustrated vocalizations. Blue lines indicate group-level comparisons. Red lines indicate the effect size patterns of individual subjects to highlight variability across subjects. The first two columns look at the similarities in effect size patterns for the movement of formants, which show higher complexity in delight, request, and self-talk vocalizations as compared to dysregulated and frustrated vocalizations. The second two columns look at the similarities in effect size patterns for F0, which show lower complexity in delight, request, and self-talk vocalizations as compared to dysregulated and frustrated vocalizations

**Fig. 8:**
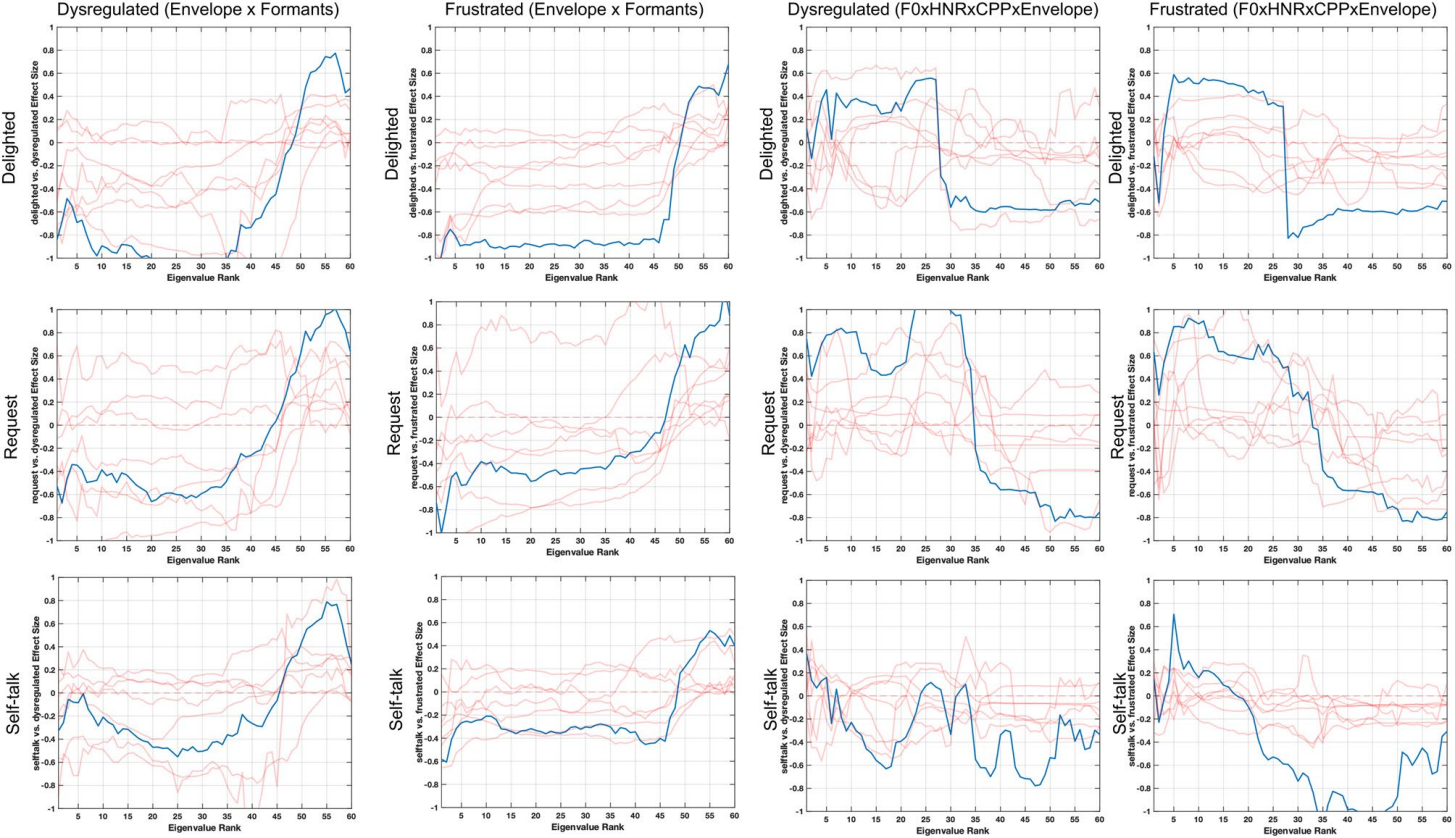
Cohen’s d effect sizes comparing eigenvalues from delight, request, and self-talk vocalizations to dysregulated and frustrated vocalizations. Blue lines indicate group-level comparisons. Red lines indicate the effect size patterns of individual subjects to highlight variability across subjects. The first two columns look at the similarities in effect size patterns for the interactions between speech envelope and formants, which show higher complexity in the first three categories. The second two columns look at the similarities in effect size patterns for interactions between F0, HNR, CPP, and speech envelope, which show lower complexity in delight, request, and self-talk as compared to dysregulation and frustration

Though there are some differences in the complexity of speech production movements between frustrated and dysregulated vocalization, there are many similarities as well. Some differences and similarities are seen in comparing each of the remaining three vocalization categories—self-talk, request, and delight—with frustrated and dysregulated. Figure 6 depicts spectrograms that highlight differences in request and dysregulation vocalizations for a single subject (P05), showcasing a higher degree of laryngeal modulation in the dysregulated vocalization (bottom) as compared to the request vocalization (top). Figures 7 and 8 highlight features with clear effect size patterns for the comparison of delighted, request, and self-talk as compared to frustrated or dysregulated for two sets of feature combinations each.

In Fig. 7, we focus on complexity of signals within a single speech production subsystem. Specifically, we find that formants, representing the articulatory subsystem, have higher complexity in delight, request, and self-talk vocalizations as compared to dysregulated and frustrated, due to the negative-to-positive effect size pattern. In contrast, F0 trajectories show lower complexity in delight, request, and self-talk vocalizations as compared to dysregulated and frustrated vocalizations, as seen by the positive-to-negative effect size pattern. Figure 8 focuses on correlations across subsystems for two combinations of features. The first, envelope x formants, represents the interaction between the respiratory and articulatory subsystems, and highlights that across subjects and the categories, delighted, request, and self-talk vocalizations have higher complexity of the coordination of articulatory and respiratory movements as compared to those of frustrated or dysregulated vocalizations. The second feature combination, F0 × HNR × CPP × envelope represents the interaction between the laryngeal and respiratory subsystems, and indicates that delighted, request, and self-talk vocalizations have lower complexity in the coordination of movements across the two subsystems as compared to frustrated or dysregulated vocalizations, though the pattern is strongest in the delighted and request comparisons.

These results suggest that the distinction between these two groups (frustrated or dysregulated versus delight, request, or self-talk) is dominated by differences in two patterns: higher complexity in the coordination of respiration x articulation and lower complexity in the coordination of respiration × phonation.

### Self-Talk vs. Request vs. Delight

**Fig. 9:**
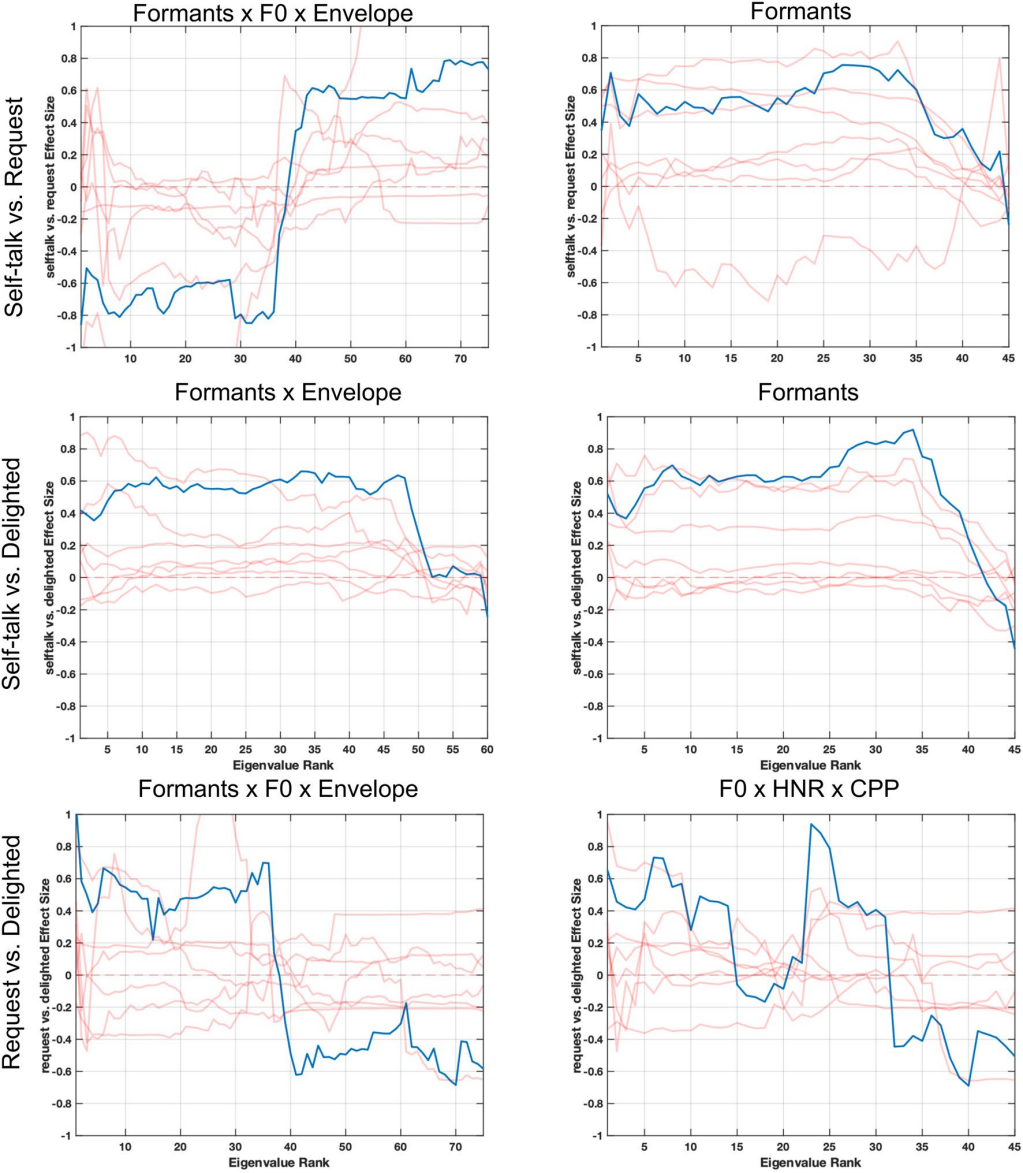
Cohen’s d effect size patterns across eigenvalue comparisons between self-talk, request, and delight vocalizations. Blue lines indicate group-level comparisons. Red lines indicate the effect size patterns of individual subjects. There are differences observed in cross-subsystem correlations between each combination of features. Specifically, request seems to have lower complexity of the coordination across all three speech production subsystems

We separated the comparison between these three categories from frustrated and dysregulated as they capture different emotions and communicative behavior. Figure 9 shows the effect size patterns from coordination across subsystems as well as the coordination within a subsystem that showed clear effect size patterns when comparing the three categories. When comparing self-talk vocalizations with request vocalizations, we find that differences exist in the interaction between movements in all three subsystems. Formant trajectories on their own show lower complexity in self-talk as compared to request vocalizations. Yet, the interaction of formant, speech envelope, and F0 movements show higher complexity in self-talk vocalizations as compared to request vocalizations. When comparing self-talk vocalizations vs. delighted vocalizations, we find that self-talk vocalizations have lower complexity of both formant trajectories as well as the interaction between formant and speech envelope trajectories. Finally, in comparing request and delighted vocalizations, we find that the interactions across all three speech subsystems, with formant, F0, and envelope trajectories, show lower complexity in the request vocalizations as compared to the delighted vocalizations. Although not as distinct, this is also seen in the interaction within the laryngeal subsystem, with lower complexity seen in the correlation of F0, HNR, and CPP trajectories (Fig. 9).

## Discussion

This paper presents a unique analysis of categories of vocalizations from a database of vocalizations from non- or minimally-speaking individuals in real-world environments. The categories—frustration, dysregulation, request, self-talk, and delight—can be interpreted by caregivers of non- or minimally-speaking individuals, but it can be hard for a new listener to discern between them. Correlation-based features, representing the coordination within and across three speech subsystems—articulatory, laryngeal, and respiratory—were used to identify the types of changes that non- or minimally-speaking individuals may make in movements of their speech production subsystems to differentiate between the vocalization categories. This work provides insight into both the acoustic differences between the categories as well as the localization of how those acoustic differences may arise. The results from this paper could be used to develop a curated feature set to provide interpretability of vocalizations from non- or minimally-speaking individuals and also to better understand how motor coordination and communication are linked in speech production. The insights could additionally be utilized by clinicians to better understand how speech production movements differ across vocalization classes, and how they could be modified to better communicate the desired intent.

### Vocalization Analysis

The frustrated and dysregulated categories did not show many clear differences between movement and coordination across and within speech subsystems, other than in the laryngeal subsystem. Consistent with spectrograms, the frustrated category appeared to have lower complexity of movement, with less change in frequency of vocal fold vibration (F0) and irregularity of this vibration (CPP) over time as compared to vocalizations from the dysregulated category, as shown in Fig. 5. This indicates that individuals don’t modulate their pitch (vocal fold vibration) as frequently during frustration utterances as compared to during dysregulated utterances.

Subsequently, it was found that there was a clear division between frustrated and dysregulated vocalizations as compared to self-talk, request, and delighted vocalizations. Specifically, there appears to be lower complexity of laryngeal and respiratory movements in the self-talk, request, and delighted vocalizations in comparison to frustrated and dysregulated vocalizations, as shown in Figs. 7 and 8. This may indicate that prosodic information, typically modulated by the laryngeal subsystem, may help in distinguishing frustrated and dysregulated states from these other categories. Perceptually, this may manifest as rapid changes in loudness and pitch during frustration and dysregulation as compared to the other vocalization classes. On the other hand, there is higher complexity of articulatory movements in the self-talk, request, and delighted vocalizations as compared to frustration and dysregulation, as shown in the spectrograms in Fig. 6 and effect size patterns in Fig. 7, which highlights that articulatory information is perhaps not used to communicate frustrated and dysregulated states for these participants. Individuals are likely utilizing rapid articulatory movements to portray self-talk, request, and delighted vocalizations.

Request vocalizations had lower complexity in the correlation across signals from all three subsystems as compared to self-talk and delighted vocalizations, as shown in Fig. 8. Combined with the comparison to frustrated and dysregulated vocalizations, this suggests that simple, coupled movements may be used in the respiratory and laryngeal subsystems by non- or minimally-speaking individuals to ask for something from the individuals around them, and they include articulatory movements to further distinguish their intent from frustrated and dysregulated states. These observations match with perceptual observations, where there was typically more consonants in request sounds, while frustrated and dysregulated vocalizations included exclamations, squeals, and content that sounded more like a sustained vowel, such as holding the vowel ‘a’ for a period of time. On the other hand, delighted vocalizations appeared to have the highest complexity of movements across all three subsystems as compared to request and self-talk, which may indicate that there is less control over coordination of movements across the speech production subsystems during the expression of this vocal categorization.

### Limitations, Future Work, and Clinical Implications

Many of the tools and algorithms we utilized for low-level acoustic feature extraction have been developed and utilized for speech recordings in controlled environments, and have typically been utilized in feature extraction in verbal communication. Therefore, there may be some noise in the low-level features extracted, as recordings in this study were done in a real-world environment, with possible background noise, varied recording conditions, and a focus on non-verbal vocalizations. In future work, we will explore noise-reduction techniques and further evaluate parameters of the feature extraction techniques to increase robustness of feature extraction.

In the analysis presented here, we eliminated the ‘social exchange’ vocalization category due to the low number of labeled occurrences across all of the subjects. Moving forward, it will be important to emphasize the importance of collecting examples from this category, as social exchanges can help in better understanding communication differences across non- or minimally-speaking individuals. Within the social exchange category and across all other vocalization categories, we may also be able to understand changes made in speech production subsystems when interacting with a caregiver vs. a peer vs. a clinician, which would be important to incorporate into our understanding of how non- or minimally-speaking individuals communicate with different individuals. Given a potential overlap between the social exchange category and other categories, we can gain insight into how the acoustics of social exchanges alter depending on additional vocalization categories and contexts. In addition, the current analysis relies on the labeling from the caregiver, but incorporating labels from clinicians, teachers, and other individuals may also aid in developing personalized models and insights to understand how non-verbal vocalizations change with respect to social context.

Caregivers were instructed to label vocalizations when they were certain that there was a single communicative function or emotion being conveyed with that vocalization. However, this excludes the possibility that there are overlapping functions and emotions in vocalizations, such as ‘dysregulated request’ or ‘delighted self-talk’, which would be important to identify for the individual. In addition, there could be vocalizations that sound very similar to each other acoustically, but were interpreted as different communicative intents given the context in which they were produced. In initial versions of the application, the introduction of multiple labels for vocalizations was overwhelming for the labeler, which led to the instructions used in the data collection for this study. This labeling procedure was replaced with the application asking for caregivers to only label a vocalization when they were confident in the meaning and intent, given the contextual clues, which greatly reduced the cognitive load of the labeling procedure and made it much easier for the caregivers to provide the data included in this study. Being the first of its kind to label the vocalizations of non- or minimally-speaking individuals in the wild, we strove to reduce cognitive load and interference with daily life for the caregiver in the current study, thus allowing for a large number of collected utterances from each individual. However, moving forward, we will test out application interfaces that will enable for multiple labels such that we can get more accurate analysis of vocalizations with multiple communicative intents. We will also present the opportunity for multiple caregivers to participate in labeling at the same time so that we are able to establish inter-rater reliability and determine how consistent the labels are for each individual across caregivers.

The current analysis focuses on the vocalizations of 7 individuals, but given the variability across the population of non- or minimally-speaking individuals and the different conditions that can cause an individual to be non- or minimally-speaking, there might be patterns that are stronger for some subgroups of individuals compared to the larger group. While there were many additional individuals who were interested in participating in the study, many were unable to incorporate the recording procedure into their daily lives easily. This warrants the creation of a simpler recording procedure and application, which will be as non-invasive in daily life as possible, while also collecting a large variety of vocalizations. With this simpler recording procedure, we hope to expand this study to include a larger number of individuals and conditions, such that we can verify whether the patterns observed here carry over to a larger set of individuals. In addition, we will be able to conduct subgroup level analyses within specific diagnoses to determine whether characteristics of the disorder affect these patterns, e.g., in CP vs. ASD.

We also analyzed subjects across a wide age range of 6–23 years. In that time, neurotypical individuals typically undergo physiological changes, such as lengthening of the vocal tract, as well as improved coordination of articulators. However, these changes have not yet been studied in non- or minimally-speaking individuals, where individuals have limited expressive vocabulary, and have not been analyzed in non-verbal vocalizations. As we expand the number of subjects in this study and conduct a longitudinal analysis, we aim to analyze any changes to non-verbal vocalizations that may occur as individuals age and link those changes to physiological changes in each individual. Future work will aim to increase the number of subjects so that we can compare specific subgroups—i.e., focusing on similar levels of cognitive status, and smaller age groups. We could also compare to nonverbal infants aged 0–1 years to see whether verbalization classes from infants are similar to those from non- or minimally-speaking individuals.

Additionally, while the group level analyses did highlight clear patterns that distinguished between categories, there was variation across individuals, where some individuals had patterns that were opposite to that of the group level. This highlights the need for personalized modeling of vocalizations. There are subtleties that differentiate between the types of acoustic modulations that will be used by non- or minimally-speaking individuals to communicate different intents, and these may not necessarily carry over to the next individual. For clinicians and therapists, who may be seeing multiple patients, it will be important to develop a personalized model and analysis of the vocalizations of a non- or minimally-speaking individual, which would allow for the clinician or therapist to easily interpret the intent of the vocalization. An initial set of models have been developed for some of the individuals in this study (Narain et al., 2022), and we aim to augment and further inform feature selection of the models based on the patterns that we observe in this paper. Motoric movements (both fine- and gross-motor) and gestures also contribute to a caregiver’s perception and understanding of a non-verbal vocalization and were utilized when caregivers were labelling the audio. Though we would like to continue to focus on characterizing vocalizations using audio, as this is a simple, non-invasive way of collecting data, future work may evaluate the utility of visual and environmental information to augment characterization of non-verbal vocalization categories, which may lead to improved personalized classification models.

Altogether, future work will aim to incorporate the speech-based features of this paper to develop an easy-to-use application which can interpret the vocalizations and additional contextual information in real time. This can help facilitate communication between non- or minimally-speaking individuals and the people around them who are unfamiliar with their communication patterns, such as visiting grandparents, new teachers, or new therapists. This will increase the independence and agency of non- or minimally-speaking individuals. We additionally will gain insight into the motor coordination strategies used by non- or minimally-speaking individuals for communication, and compare them to those from neurotypical individuals to get closer to understanding the link between motor coordination and communication. Finally, in clinical practice, consistent tracking of motor coordination-based features could aid in understanding the effect of therapeutics on motor coordination and communication over time, and may lead to the development of additional strategies to assist communication in non- or minimally-speaking individuals.

## Supplementary Information

Below is the link to the electronic supplementary material.

Supplementary file1 (DOCX 400 kb)
