# Supplementary material for: Brief Report: Quantifying Speech Production Coordination from Non- and Minimally-Speaking Individuals
Source: J Autism Dev Disord. 2024 Apr 13;56(3):1266–80. doi: 10.1007/s10803-023-06206-0 (PMC12935788; doi:10.1007/s10803-023-06206-0)
Supplement: Supplementary file 1 — Supplementary file1 (DOCX 400 kb) [file 10803_2023_6206_MOESM1_ESM.docx]

## Supplemental Materials: Simulations of Complexity

When comparing correlation matrices to one another, we can determine relative complexity based on comparison of the eigenspectra, and group level comparisons leading to effect size patterns. However, we additionally would like to understand what changes we can observe in the raw signals that contribute to differences in complexity. In this section, we lay out three modifications that we make to signals to change their complexity and showcase how the eigenspectra change. We present these additions in the case of both a single channel (signal) as well as a case with multiple channels and explore the hypothesis that the resulting modified signals will have higher complexity as compared to the original, reference signals.

### Noise

The first change we make is the addition of gaussian white noise to a reference signal. The sinusoid we begin with for the one channel case is:

$$y_{1}[n]= \cos(2\pi n)$$

Our time vector is constructed at 300 Hz for 3 seconds. In the multi-channel case, we add in two additional sinusoids that are the same base sinusoid shifted up vertically:

$$y_{2}[n]= \cos(2\pi n)+2$$

$$y_{3}[n]= \cos(2\pi n)+4$$

To each of these sinusoids, we add gaussian white noise with a signal-to-noise ratio (SNR) of 10. We then calculate the correlation matrices in both the single channel and multi-channel cases and extract the eigenspectra. In this case, there are 15 eigenvalues extracted from the correlation matrix in the single channel case, and 45 eigenvalues extracted from the correlation matrix in the multi-channel case. Due to the scale of the eigenvalues, the eigenspectra are plotted on a log scale, ranked from greatest to smallest. Figure S1 shows the eigenspectra from the single channel and multi-channel simulations, with and without gaussian white noise added.


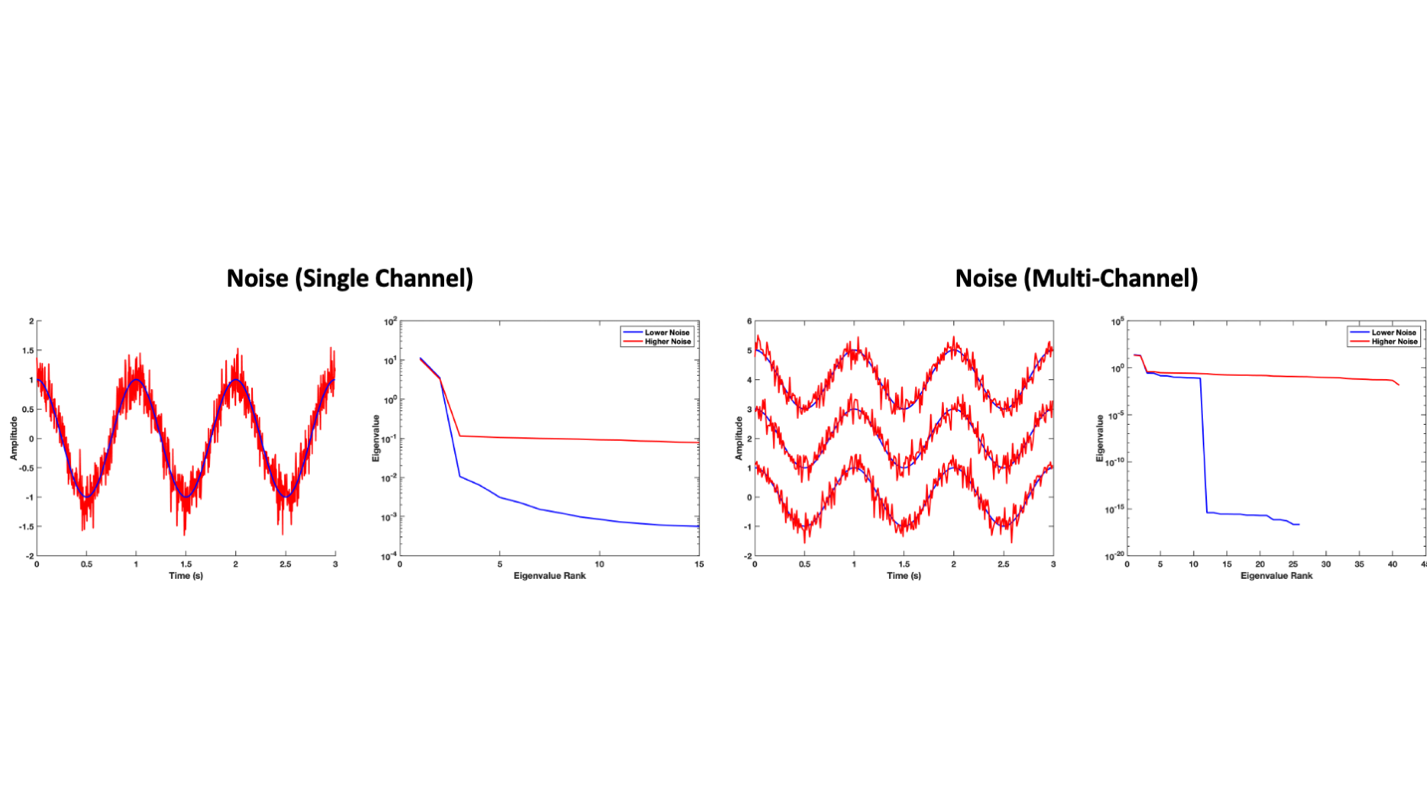


Figure S1: Signals with and without gaussian white noise added. On the left, we present the single channel case, and on the right we present the multi-channel case. In both cases, the addition of noise (in red) causes eigenvalues from the resulting correlation matrix to be more uniformly spread out as compared to the case without noise (in blue). This indicates that the addition of noise leads to higher complexity in the auto- and cross-correlations of signals.

In Figure S1, the original sinusoids are shown in blue and the sinusoids with noise added are in red. As we can see in the resulting eigenspectra, the eigenspectra from the noisy signals starts off at a lower magnitude as compared to the eigenspectra from the base signals, but has a higher magnitude in higher ranked eigenvalues. Referring back to Figure 3 from the main text, this indicates that the signals with noise added, in both the single channel and multi-channel cases, have higher complexity as compared to the base signals.

### Higher frequency content

The next simulation tests how the eigenspectra change when signals are a higher frequency as compared to a base signal. We begin with the same time vector, lasting 3 seconds at 300 Hz. In the single channel case, we start with an original signal and modified signal of:

$$y_{\mathrm{original}}[n]= \cos(0.5\pi n)$$

$$y_{\mathrm{modified}}[n]= \cos(2\pi n)$$

And in the multi-channel case, we have the original signals:

$$y_{1,original}[n]= \cos(2\pi n)$$

$$y_{2,original}[n]= \cos(2\pi n)+2$$

$$y_{3,original}[n]= \cos(2\pi n)+4$$

And in the modified case, we change the frequencies of the 2^nd^ and 3^rd^ signals to be higher:

$$y_{1,modified}[n]= \cos(2\pi n)$$

$$y_{2,modified}[n]= \cos(4\pi n)+2$$

$$y_{3,modified}[n]= \cos(8\pi n)+4$$

The plots of these signals and their resulting eigenspectra are in Figure S2.


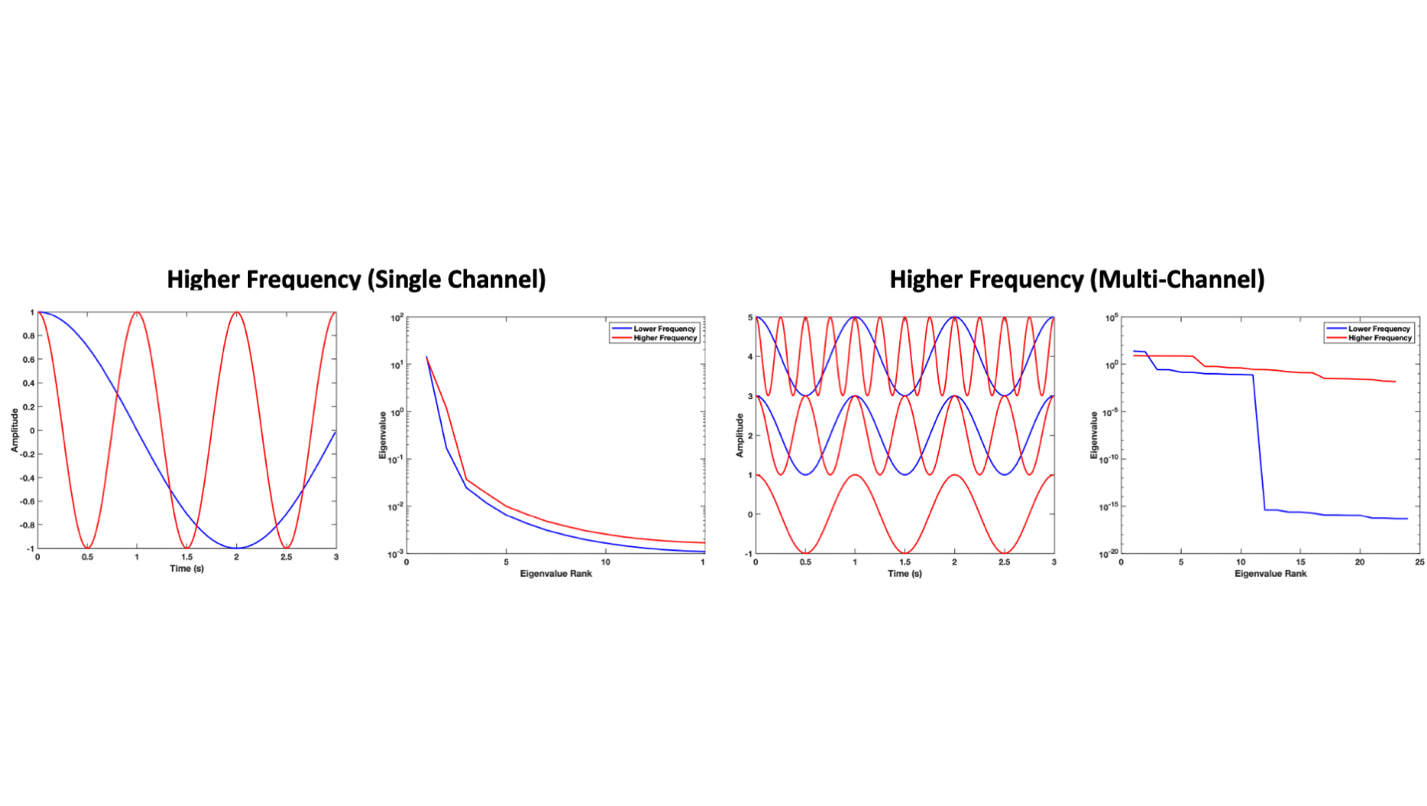


Figure S2: Depiction of the difference in eigenvalues when signals of varying frequencies are compared. In both in the single channel and multi-channel case, higher frequency signals (red) have higher complexity as compared to signals with lower frequency (blue), as the resulting eigenvalues from the correlation matrix are more evenly distributed.

As hypothesized, the higher frequency signals (in red) show higher complexity through the spread of their eigenvalues as compared to the base signals (in blue). This appears in both the single channel and multi-channel case, highlighting that the complexity can arise from both auto- and cross-correlation differences when the signals have higher frequency content.

### Phase Shift

The final simulation only applies to the multi-channel case, and simulates the channels undergoing a phase shift relative to each other. Many motoric actions depend on certain coupling of movements, and changes in those relative timings of the movements could cause issues in completing an action (e.g. moving your eyes together at the same time to look at an approaching target). Once again, we generate a time vector lasting 3 seconds at 300 Hz, and create correlation matrices. The original signals are the same as before in the multi-channel cases:

$$y_{1,original}[n]= \cos(2\pi n)$$

$$y_{2,original}[n]= \cos(2\pi n)+2$$

$$y_{3,original}[n]= \cos(2\pi n)+4$$

In the modified cases, we generate a phase shift for the 2^nd^ and 3^rd^ signals:

$$y_{1,phase\_shift}[n]= \cos(2\pi n)$$

$$y_{2,phase\_shift}[n]= \cos(2\pi n+100)+2$$

$$y_{3,phase\_shift}[n]= \cos(2\pi n+200)+4$$

The signals and their resulting eigenspectra are plotted in Figure S3.


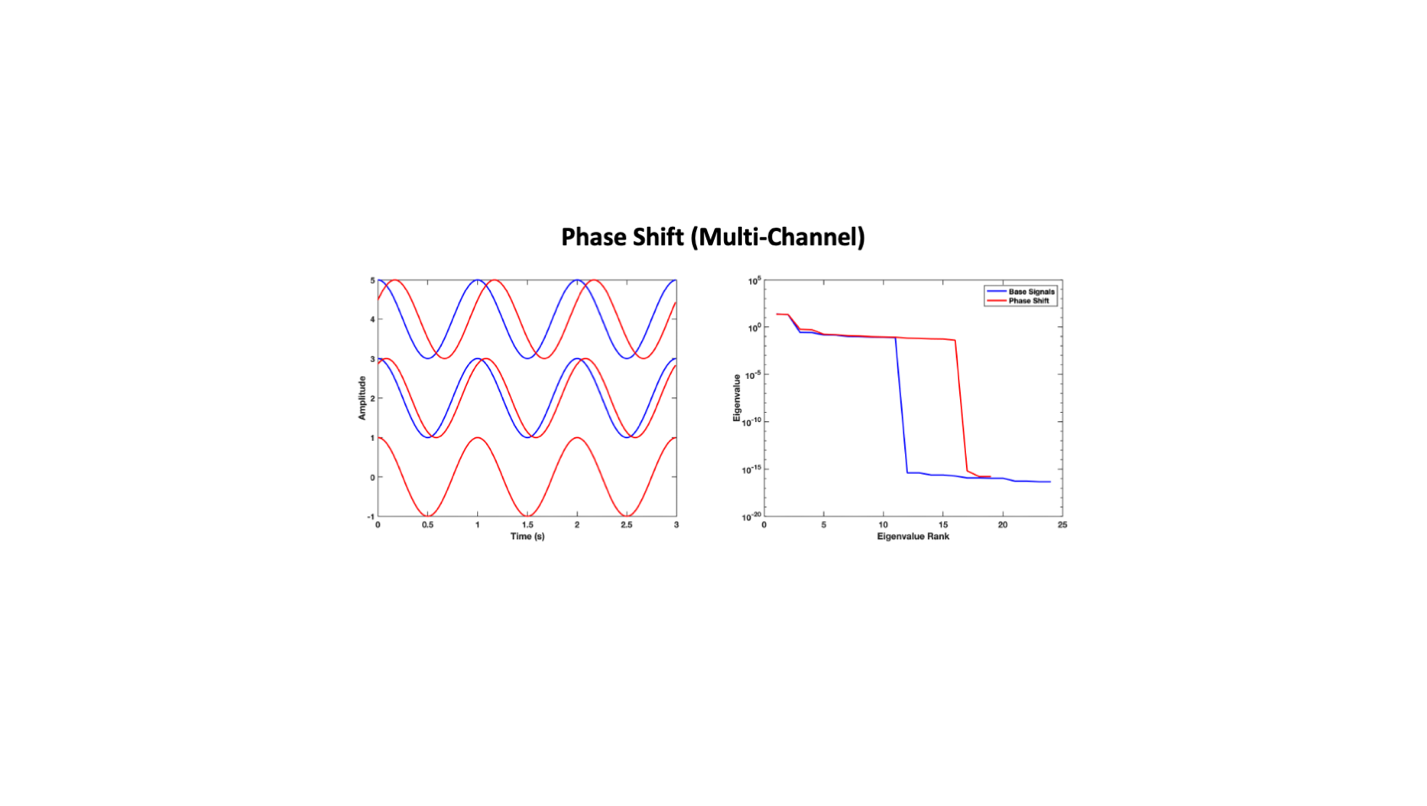


Figure S3: Depiction of signals and resulting eigenvalues for a case where the signals are all coupled (in blue) versus the case where signals are shifted in phase relative to each other (in red). The eigenvalues from the phase shifted case show that the signals have higher complexity as compared to the case where the signals are in phase with each other.

We can see that the addition of the phase shift, based on the signals in red, causes the eigenspectra to show higher complexity as compared to the original signals. Altogether, these simulations help demonstrate the changes that can occur to the acoustic and articulatory time series that we analyze in the main manuscript that lead to the differences in complexity between vocalization classes.
